# Dry Gangrene of the Penis Induced by a Bullring for Sexual Stimulation Purposes

**DOI:** 10.1100/tsw.2008.128

**Published:** 2008-09-21

**Authors:** Apostolos P. Labanaris, Vahudin Zugor, Robert Smiszek, Reinhold Nützel, Reinhard Kühn

**Affiliations:** ^1^ Department of Urology, Martha Maria Medical Center, Nurnberg, Germany; ^2^ Department of Urology, Salzgitter Medical Center, Salzgitter, Germany

**Keywords:** penile strangulation, ischemia, dry gangrene, therapeutic approach

## Abstract

Dry gangrene of the penis is a critical clinical condition provoked by vascular compromise that can lead to severe complications. Although usually caused by diabetes mellitus or due to end-stage renal disease, in these last years, there has been an increase of penile gangrene incidents induced by penile strangulation due to constricting devices for sexual stimulation purposes. We present such a case and discuss the correct approach for treatment of this unusual condition.

## INTRODUCTION

The attachment of constricting devices to the penis is used by some men for sexual stimulation and in an effort to increase their sexual performance, since strangulation of the penis can increase sexual interest and can also result in a longer erection. Attaching such devices on the semi-erected penis often leads to the impossibility of their removal after erection. As a result, not only mechanical injuries can be caused, but also severe vascular complications, such as vascular insult, ischemia, and dry gangrene of the penis. Dry gangrene of the penis is a rare emergency situation that requires urgent intervention in order to alleviate penile pain due to ischemia, as well as to improve the quality of life of the patient in regard to wound care, sexual function, and lower urinary tract management.

## CASE REPORT

A 58-year-old patient presented with dry gangrene of the distal penis, comprising the glans and a part of the shaft. He explained that 5 days previously, a bullring had been placed over his external genitalia. The bullring was left in place for 3 days and then removed with a bolt cutter. The patient reported that the day the bullring was removed, the distal one-third of the penis had begun to turn black. He also admitted that it took him 2 days to present to us, not only because his pain had just begun, but also because was feeling ashamed about this uncomfortable incident. The patient had consulted with his urologist and decided to present to our department. He mentioned that he was able to void spontaneously and did not exhibit any signs of dysuria, hematuria, or fever. His medical history revealed Parkinson's disease, but no history of nicotine, alcohol abuse, peripheral arterial disease, or diabetes mellitus. Clinical examination revealed a patient in distress and in pain; the penis showed dry gangrene of the distal one-third, including the glans and penile shaft skin extending about 1.5 cm proximal to the coronary sulcus (Fig. 1). The necrosis was sharply demarcated and although a putrid odor was present, no signs of infection were identified. The penile shaft was slightly swollen, but not tender on palpation. On sonography, the bladder, although previously emptied as reported by the patient, contained about 200 ml residual urine. Both testicles were normal.

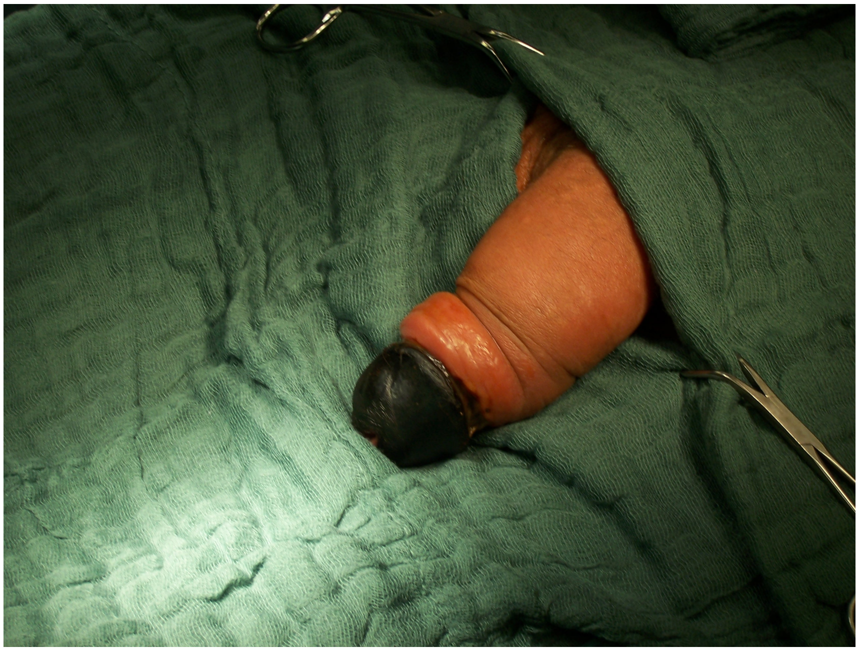

**FIGURE 1.** Preoperative view exhibiting dry gangrene of the glans with involvement of the distal shaft.

Urinalysis exhibited 500 leucocytes with positive nitrates. The urine culture exhibited a growth of *Escherichia coli* and *Enterococcus*, both of which were sensible to Ampenicillin. The laboratory values showed only an elevated white blood count of 13/nl.

The patient was given antibiotic prophylaxis and then a partial penectomy was planned. At first, a urethrocystoscopy was performed in order to estimate the length of urethral involvement, then a partial penectomy took place. Only macroscopic tissue necrosis was dissected up to the length of urethral involvement in order to save as much living tissue as possible. Intraoperative findings exhibited ischemic necrosis of the glans and partial necrosis of the proximal spongiosal and cavernosal corporal tissues (Figs. 2 and 3). Histopathogical evaluation indicated dry gangrene of the distal penis. The postoperative convalescence was uneventful, and the cosmetic and functional results 3 months later were impressive.

## DISCUSSION

Dry gangrene of the penis is a consequence of vascular compromise. The progressive vaso-occlusive changes that result in penile gangrene are usually caused by diabetes mellitus, a buildup of calcium deposits owing to end-stage renal disease, or penile strangulation due to constricting devices[1,2,3,4,5,6]. Strangulation objects can be metallic[7] or nonmetallic[3], and are used for sexual stimulation, in an attempt to improve sexual interest and to maintain a longer erection. The use of such devices varies and depends on

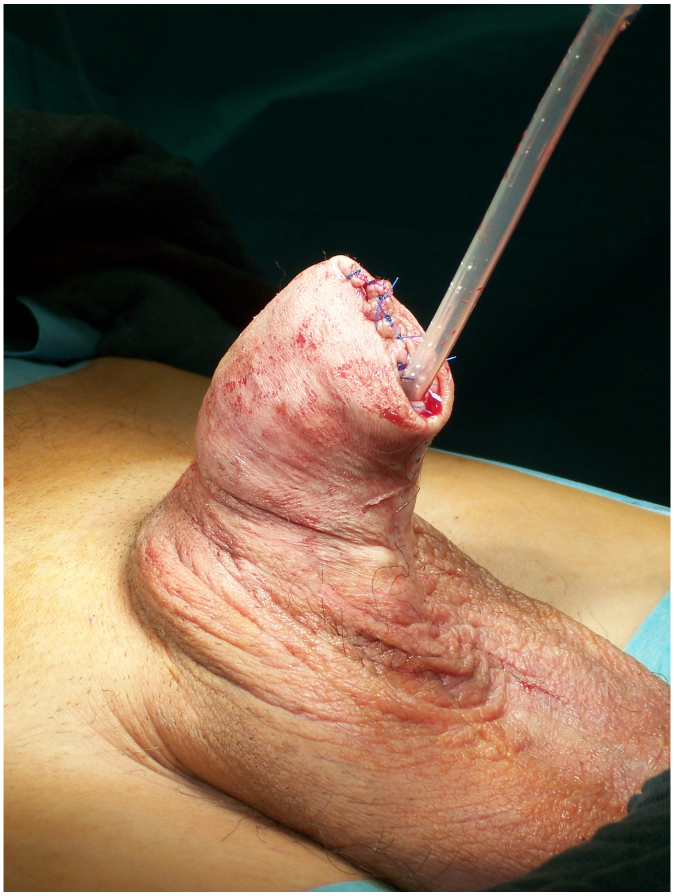

**FIGURE 2.** Postoperative view exhibiting the penis after partial penectomy.

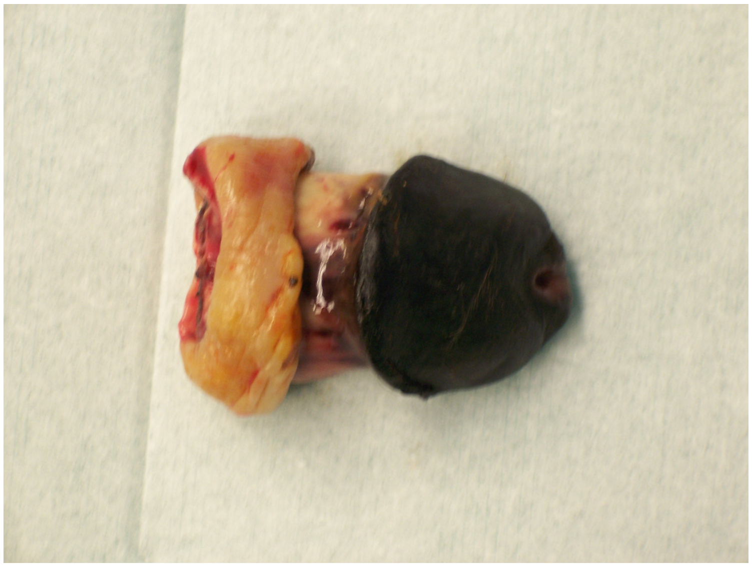

**FIGURE 3.** Partial penectomy specimen exhibiting dry gangrene of the glans and distal shaft.

the patient's age. In middle-aged and elderly men, the leading cause for their use is to increase sexual performance or due to autoerotic intentions[8], while in adolescents, use is due to the desire to increase erotic sensation during masturbation or due to sexual curiosity. Although they can increase sexual performance and sensation, using them on the semi-erect penis often results in the impossibility of their removal after erection; thus, leading from a simple penile engorgement to ulceration, necrosis, or even to severe vascular complications. Consequently, several clinical syndromes can occur: from mild nonsignificant vascular obstruction that resolves after decompression to severe penile gangrene. As the corpus spongiosum and urethra are covered by a thin layer of fibrous tissue, they are most susceptible to mechanical injury. However, gangrene is uncommon, probably because each corpus cavernosum has an individual artery, and the thickness of Buck's fascia and corporeal tissue resists pressure on the deep vessels[9]. Patients that present with dry gangrene are usually patients who have detected the problem earlier, but are feeling embarrassed about their situation, or patients who are mentally ill[10]. As seen earlier, the aforementioned patient presented to us 2 days after he noted that his distal penis had begun to turn black. He admitted that he was feeling humiliated and did not want to share his problem even with a doctor.

What is of extreme importance is the clinical differentiation between dry gangrene due to ischemic disease and wet gangrene with infection. This is crucial for choosing the appropriate treatment. Wet gangrene is infectious in origin, and prompt surgical intervention is indicated to decrease associated morbidity and mortality[10]. If left untreated, rapidly progressive tissue destruction, sepsis, and ultimately death can occur. Partial or total penectomy is the treatment of choice.

Although the causes of dry gangrene vary, treatment options are only two: partial penectomy or conservative management. Weiner and Lowe[6], reporting on a series of seven patients, found that delaying intervention usually required more extensive surgery and increased the risk of wound complications. Thus, they advocate aggressive surgical treatment initially, although they observed wound complications in one case that required several more surgeries. Simply observing these patients usually leads to subsequent liquefaction, infection, and urinary obstruction, which will necessitate surgical intervention with percutaneous suprapubic tube drainage and debridement[6].

The indication for conservative treatment usually involves small circumscribed lesions[5] or high-risk multimorbid patients with generalized arterial occlusive disease[3]. If conservative management is to be considered first, then circumcision should be at least performed (if the patient is not circumcised) in order to allow better observation of disease progression, as well as to allow dry healing. However, if the disease progresses by necrotic skin on the shaft, liquefaction, development of infection, crepitus, or induration of the shaft, then partial or total penectomy should be performed.

In conclusion, dry gangrene of the penis due to penile strangulation is a clinical condition that could lead to severe complications. What is of extreme importance is the clinical differentiation between gangrene due to ischemic disease and gangrene due to infection. Prompt diagnosis and treatment are essential to decrease associated morbidity and mortality, as well as to achieve good cosmetic and functional results.
